# Photodynamic Decontamination of Food: Assessing Surface Challenges Against *Listeria monocytogenes*

**DOI:** 10.3390/microorganisms14010059

**Published:** 2025-12-26

**Authors:** Anabel Cenit, Jun Liu, Michael Fefer, Kristjan Plaetzer

**Affiliations:** 1 Laboratory of Photodynamic Inactivation of Microorganisms, Department of Biosciences and Medical Biology, University of Salzburg, 5020 Salzburg, Austria; anabel.santiagocenit@plus.ac.at; 2 Suncor Energy Inc., Calgary, AB T2P 3E3, Canada

**Keywords:** photodynamic inactivation, food safety, *Listeria monocytogenes*, chlorophyllin

## Abstract

*Listeria monocytogenes* is a foodborne pathogen of significant concern. While it typically causes mild, self-limiting gastroenteritis, it poses a much higher threat to immunocompromised individuals and pregnant women, where it may lead to miscarriage. Numerous outbreaks have been linked to ready-to-eat foods. Although heat treatment is commonly used for microbial decontamination, it is unsuitable for fresh produce such as fruits and vegetables. Other physical (e.g., UV, gamma irradiation) and chemical (e.g., NaOCl, ozone) methods can compromise sensory qualities or face limited consumer acceptance. Photodynamic Inactivation (PDI) has emerged as a promising alternative, particularly when using natural photosensitizers. Because PDI efficacy depends on photosensitizer diffusion, there is a need to further explore how different and complex fruit surface structures may influence its performance. Three fruit models were therefore selected to represent distinct surface textures and were evaluated in situ: apples (smooth), strawberries (irregular), and kiwis (fuzzy and hairy surface). The influence of contamination order was also evaluated, as this factor is highly relevant to real-world supply-chain scenarios but has been largely overlooked in prior research. Additionally, the study investigated how the order of contamination affected the decontamination outcome. Sodium-magnesium-chlorophyllin (Na-Mg-Chl), an approved food additive (E140), was used as photosensitizer. Fruits were cut into 1 cm^2^ squares and inoculated with *L. monocytogenes*. A 100 µM Na-Mg-Chl solution was applied either before or after bacterial inoculation. All samples were then illuminated using a 395 nm LED (radiant exposure 15 J/cm^2^). When *L. monocytogenes* was applied first, followed by the addition of Na-Mg-Chl, a 5.96 log reduction was observed in apples, a 5.71 log reduction in strawberries, and a 6.02 log reduction in kiwis. Conversely, when Na-Mg-Chl was applied prior to bacterial deposition, apples showed a 5.61 log reduction, strawberries demonstrated a 6.34 log reduction, and kiwis achieved the highest inactivation, at 6.74 log units. These results indicate that PDI consistently achieved substantial bacterial reductions across all fruit types, regardless of surface characteristics or application order. This supports PDI as a powerful method for fruit surface decontamination, reducing public health risks and economic losses while preserving product quality and consumer confidence.

## 1. Introduction

*Listeria monocytogenes* is a Gram-positive bacterium that exhibits both saprophytic behavior and opportunistic pathogenicity. Its ability to survive at refrigeration temperatures and across a variety of food matrices makes it a persistent contaminant in fresh food products. Additionally, it forms biofilms, enhancing its resilience in food-processing environments and resistance to sanitation efforts [1,2,3]. Recent evidence suggests that *L. monocytogenes* is developing resistance to certain sanitizing agents. For instance, two genotypes (ST 1003 and ST 554) from fruit packing facilities have minimum inhibitory concentrations (MICs) for peroxyacetic acid that exceed routinely applied concentrations [4]. Antimicrobial resistance has also been observed, particularly against trimethoprim-sulfamethoxazole and erythromycin, although clinical resistance remains rare [1,2].

In healthy individuals, infection typically results in mild, self-limiting gastroenteritis. However, in immunocompromised populations, *L. monocytogenes* can cause invasive disease, resulting in severe outcomes such as sepsis, encephalitis, or death. Pregnant women are especially at risk, as infection may induce miscarriage. Invasive listeriosis is associated with a mortality rate of 20–30% in susceptible individuals, even with appropriate treatment [1,2,3,4,5]. These risks emphasize the critical role of well-established microbiological food safety systems. Recent listeriosis outbreaks in the United States and Europe [6,7] have highlighted the urgent need for effective decontamination methods to mitigate its presence in food products [8]. In the United States alone, the economic burden of foodborne illnesses, including those caused by *L. monocytogenes*, is estimated at approximately USD 75 billion annually, accounting for medical expenses, lost productivity, and other associated costs [9]. In Europe, listeriosis is the second most frequent cause of foodborne infection-related deaths after salmonellosis, resulting in high fatality rates [5]. Among various food categories, ready-to-eat (RTE) products, particularly fresh produce such as vegetables and fruits, present a higher risk because they are typically consumed without prior heat treatment and are therefore more susceptible to cross-contamination [2,3].

Some of the most widely used measures to decontaminate food in the EU are heat-based methods [10], physical methods, and chemical methods [11,12]. All of these have advantages and disadvantages in terms of fruit decontamination, but thermal methods can adversely affect fruit tissue. Chemical approaches, such as the use of sodium hypochlorite (NaOCl) or ozone, can help kill harmful microorganisms on fruits and vegetables, but they can also form dangerous chloro-organic by-products such as trihalomethanes and chlorates, while ozone can produce aldehydes, ketones, and bromates. Several of these by-products have been associated with carcinogenic and mutagenic effects [13,14]. Additionally, ozone’s strong oxidative action can degrade valuable antioxidant compounds such as anthocyanins, with reported losses exceeding 90% in certain fruit juices, potentially reducing the nutritional quality of the final product [15]. On the other hand, among the physical methods, gamma and UV irradiation are gaining popularity; however, at higher doses, they can change food properties and develop an unpleasant odor or flavor. As a result, many food industries are exploring alternative preservation techniques, such as natural preservatives and innovative packaging solutions, which maintain product integrity without compromising consumer trust and minimize costs [16].

Photodynamic Inactivation (PDI) represents a novel treatment that could add to traditional decontamination methods. Three elements are essential in PDI: light, a photosensitizer (PS), and oxygen. When a PS is exposed to light of a specific wavelength, it absorbs the energy and enters an excited state. In this state, the PS can undergo either electron transfer (Type I photochemical reaction), producing free radicals like superoxide (O_2_^−•^) and hydroxyl radicals, or energy transfer (Type II photochemical reaction), mainly generating singlet oxygen (^1^O_2_). All these reactive oxygen species (ROS) can attack and damage microbial cell membranes, proteins, nucleic acids, and biofilms, making the process very effective against a wide range of pathogens and reducing the chances for microorganisms to develop resistance [17,18,19]. In the present study, the application of PDI is directed specifically toward food matrices, and the term photodynamic decontamination of food (PDc) is used to denote PDI-based inactivation of microorganisms on food surfaces.

**Figure 1:**
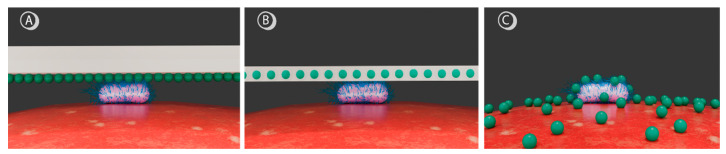
Schematic representation of different PS application strategies for food decontamination. Green spheres depict PS molecules, the rod-shaped structure represents a *L. monocytogenes* bacterium, and the red surface illustrates a fruit. (**A**) Surface adherence of PSs to packaging, (**B**) covalent binding of PSs within the packaging matrix, and (**C**) direct deposition of PSs onto the fruit surface.

In principle, three approaches can be chosen when decontaminating (packaged) food. The PS can either be adhered to the packaging materials (Figure 1A) or covalently bound to the packaging matrices (Figure 1B). As an alternative, the photoactive compound may be directly applied to food products, e.g., by spraying (Figure 1C).

The surface adherence of PSs to packaging offers a simple and versatile procedure suitable for various packaging types, with the advantage of direct PS localization in target microorganisms. However, this method requires direct contact between packaging and cells, necessitates the use of edible PSs, and may result in packaging degradation, exhaustion of the coating, and unwanted coloring. Incorporating the PS into the packaging matrix inhibits PS migration into food, allowing for a broader selection of PSs without strict biocompatibility concerns. Nevertheless, this approach is likely hampered by the limited diffusion of ROS through the material into cells and again poses risks of packaging degradation, as well as potential coloring issues.

In contrast, applying the PS directly onto food avoids packaging modifications and ensures uniform treatment, which is advantageous for irregular food geometries. However, since the PS will be incorporated into the food, edible PSs are required. In such cases, food additives are targeted, with some of the most widely used being curcumin (E100) and chlorophyllin (E140) [20]. Chlorophyllin-based PSs have shown higher efficacy against Gram-positive than Gram-negative pathogens. This could be attributed to the negative charge of chlorophyllin and the thicker peptidoglycan layer that Gram-positives have, facilitating a better attachment to the cell wall [19]. Other studies have shown that magnesium chlorophyllin has a similar singlet oxygen production to Rose Bengal and chlorin 6, making it highly suitable for antimicrobial applications [21].

In the case that the PS is directly applied, the surface structure of the food might influence the decontamination effectiveness of PDI. Therefore, the aim of this study was to compare the decontamination effect of PDI based on sodium-magnesium-chlorophyllin (Na-Mg-Chl) against *L. monocytogenes* on three types of food with different structures: apples (smooth surface), strawberries (uneven surface), and kiwis (fuzzy surface). In addition, two different decontamination approaches were tested. The inoculation of bacteria onto the surfaces before adding Na-Mg-Chl simulates the photodecontamination of food after bacteria are present; PS coatings were examined by first adding Na-Mg-Chl and then *L. monocytogenes*.

## 2. Materials and Methods

### 2.1. Cell Culture

*Listeria monocytogenes* (MRL-24-00483) was kindly provided by Ariane Pietzka (AGES, Graz, Austria). It was stored as a cryoculture, with 10% glycerol as a cryoprotective agent. Bacteria were grown overnight in a Brain-Heart-Infusion medium (Carl Roth GmbH & Co., Karlsruhe, Germany) under constant agitation (200 rpm) using a shaker incubator (MaxQ 4450, Thermo Scientific, Marietta, OH, USA) at 37 °C.

### 2.2. Photosensitizer Preparation

Sodium-magnesium-chlorophyllin (Carl Roth GmbH & Co., Karlsruhe, Germany) stock solution was prepared at a concentration of 10 mM in ddH_2_O and stored at −20 °C in the dark until use. For each experiment, it was diluted to 100 µM in Dulbecco’s Phosphate Buffered Saline (DPBS, Sigma Life Science, St. Louis, MO, USA). The concentration of Na-Mg-Chl used was selected based on preliminary decontamination efficacy trials.

### 2.3. Sample Preparation

Organic-grade apples, strawberries, and kiwis were purchased from local supermarkets and visually inspected to ensure the absence of physical damage or deterioration. For each fruit type, six biological replicates were prepared. To remove native microorganisms, all samples were sterilized with 3% NaOCl (Carl Roth GmbH & Co., Karlsruhe, Germany). Optimal decontamination time was established through preliminary testing: 1 min for strawberries and apples and 5 min for kiwis. No significant changes in color, shape, or surface characteristics were observed after treatment.

Samples were thoroughly rinsed with double-distilled water (ddH_2_O) to ensure the removal of residual NaOCl. For strawberries, the fruit was sectioned into 1 cm^2^ surface plugs using a pre-flamed scalpel. For kiwis and apples, the skin was removed prior to the disinfection process of NaOCl, after which the peeled flesh was rinsed and cut into 1 cm^2^ plugs. Prepared samples were air-dried in Petri dishes under a laminar flow hood and subsequently transferred to 24-well plates (one plug per well) (Greiner BioOne, Kremsmünster, Austria) containing 1.5% agar (Carl Roth GmbH & Co., Karlsruhe, Germany).

### 2.4. Light Source

The light source was a custom-built LED array made up of 480 LEDs that emitted primarily at 395 nm (Kingbright Electronic Europe GmbH, Issum, Germany). For the experiment, the array was mounted above the samples, which were exposed to an irradiance of 15 J cm^−2^. This fluence was measured using a LI-180 Spectrometer (LI-COR Environmental GmbH, Lincoln, NE, USA).

### 2.5. Layering Technique and Photodynamic Inactivation

**Figure 2:**
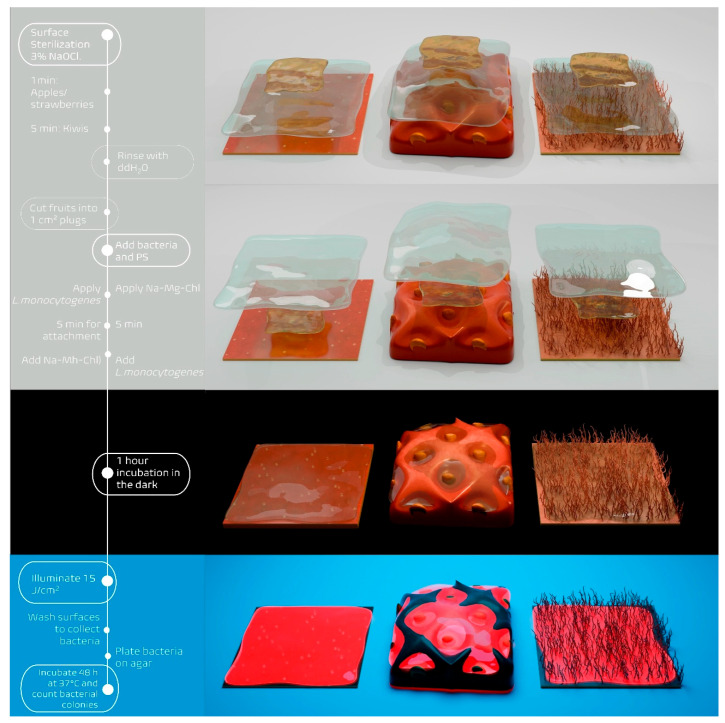
Schematic overview of the Photodynamic Decontamination experimental method. The timeline on the left shows the key steps in the process, from surface sterilization through bacterial counting. The right side illustrates the different deposition approaches and treatment phases: (**top**) PS (Na-Mg-Chl) applied before *L. monocytogenes*, (**second**) *L. monocytogenes* applied before Na-Mg-Chl, (**third**) incubation in the dark for 1 h, and (**bottom**) irradiation phase with 395 nm LED array (15 J cm^−2^). Fruit plugs (1 cm^2^) with different surface types are shown throughout: apple (smooth), strawberry (uneven), and kiwi (fuzzy).

Two approaches were employed to prepare the coated fruit surfaces containing both the PS and *L. monocytogenes,* as illustrated in Figure 2. The fruit plugs were placed in 24-well plates, with one plug per well. On each plug, 10 µL of an overnight culture of *L. monocytogenes,* with a final concentration of 10^5^–10^6^ cells/cm^2^, were pipetted. After a 5 min attachment period at room temperature, 50 µL of Na-Mg-Chl (100 µM) or, in the case of controls, DPBS, was gently applied atop the bacterial suspension to avoid disrupting the droplet surface. For each fruit type, both sequences of application were tested: either the PS or DPBS was applied before the bacteria, or the bacteria were added first, followed by the PS or DPBS.

For each experimental condition, two 24-well plates were prepared in parallel: one for light exposure and one as a dark control. Each plate contained the same treatments, including samples with the PS and *L. monocytogenes*, as well as control wells with only bacteria. After a 1 h incubation at room temperature in the dark, one plate was exposed to illumination (395 nm, 15 J cm^−2^) from above, while the other remained in the dark.

Following illumination, each plug was washed with 500 µL DPBS to dislodge bacteria. The resulting suspensions were transferred to 96-well plates, serially diluted, and plated onto HBI agar plates. Plates were incubated for 48 h at 37 °C.

### 2.6. Data Analysis

The photokilling effect was evaluated by counting colony-forming units (CFUs). Relative inactivation was calculated for each biological replicate by dividing the CFU count of the double negative control by the CFU of the corresponding treated sample [20]. Six biological replicates were conducted for each fruit type. Mean values and standard deviations were calculated across replicates. In accordance with the guidelines of the American Society for Microbiology, an antibacterial effect is considered significant when the reduction in viable cells exceeds 3 logarithmic units, corresponding to a 99.9% decrease in CFU [22,23].

## 3. Results

All numerical data are provided in the Supplementary Materials (Tables S1–S6).

### 3.1. Light Source

**Figure 3:**
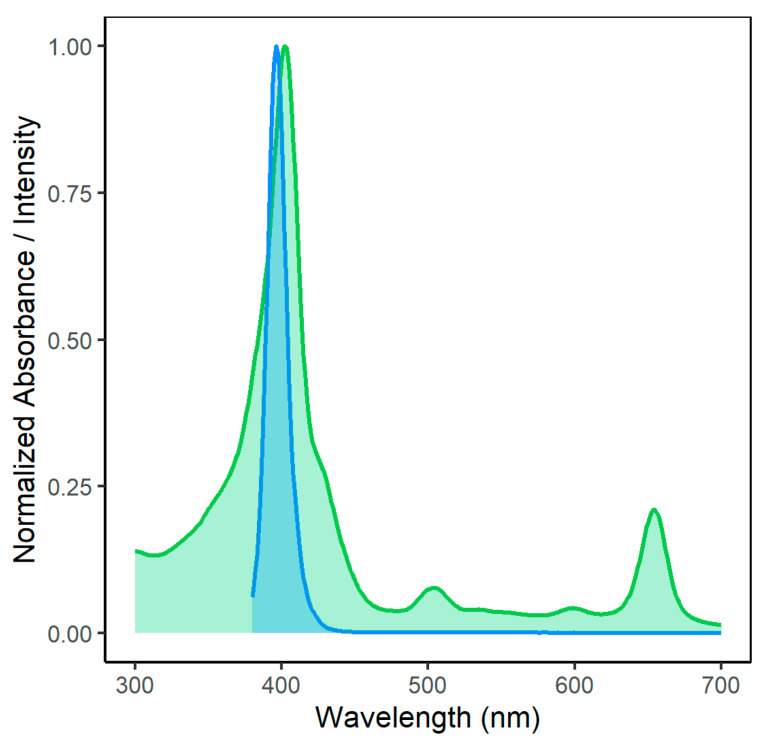
Spectral overlap between homemade LED array emission (blue curve) and Na-Mg-Chl absorbance (green curve). The absorbance signal of Na-Mg-Chl was measured using an Infinite M200 (Tecan Austria GmbH, Grödig, Austria) microplate reader. The homemade LED light source consisted of 480 LEDs, with a dominant wavelength of 395 nm (Kingbright Electronic Europe GmbH, Issum, Germany). For the experiment, samples were illuminated from above at a radiant exposure of 15 J cm^−2^. The emission of the LED source at 395 nm substantially overlaps with the Na-Mg-Chl absorbance peak at 402 nm, enabling efficient excitation.

Figure 3 shows the overlap of the emission spectrum of the light source employed in this study and the absorption of Na-Mg-Chl. For activation of the photoactive compound, the Soret absorption peak of Na-Mg-Chl at 402 nm was used.

### 3.2. Photodynamic Decontamination of Smooth Surfaces: Apples

**Figure 4:**
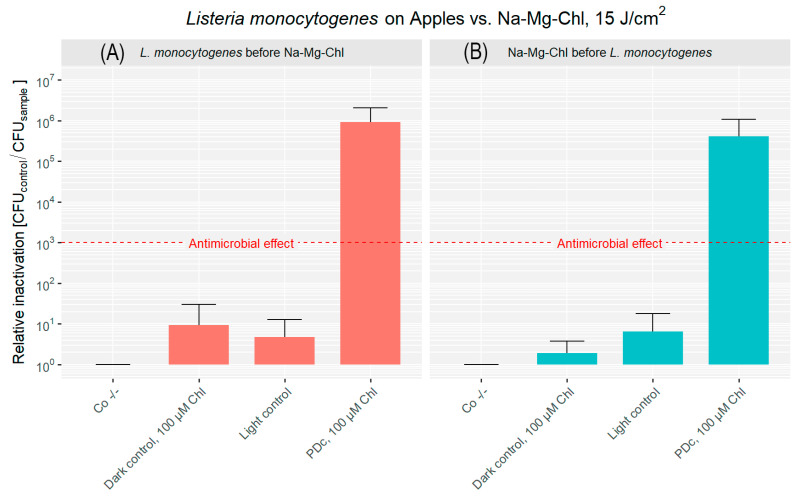
Relative inactivation (CFU_Co −/−_/CFU_sample_) of *L. monocytogenes* after PDc of apples. In (**A**), *L. monocytogenes* was applied to the apple surface first, followed by the addition of Na-Mg-Chl after 5 min. (**B**) shows Na-Mg-Chl inoculated first, with *L. monocytogenes* added onto the surface after 5 min. PDc was performed using an LED array (395 nm, 17 mW cm^−2^, 15 J cm^−2^). Controls: Co −/− (no light, no PS), Dark control (no light, PS). The light control contained DPBS and was illuminated. PDc was a positive control with 100 µM Na-Mg-Chl (Chl on the graph) and was illuminated. The red dash corresponds to a 3 log reduction. The bars represent the average of six independent biological replicates, including their corresponding error bars.

Figure 4A shows the photodecontamination efficacy for apples when bacteria were applied before the PS. The untreated controls of the experiment performed on apples had a bacterial load of 4.51 × 10^7^ CFU cm^−2^. The dark control is below 1 log reduction, while the light control shows even less bacterial reduction. The combination of Na-Mg-Chl and blue light (395 nm, 17 mW cm^−2^, 15 J cm^−2^) caused a 5.96 log reduction of the bacterial population, exceeding the antibacterial effect (99.9% reduction).

Figure 4B illustrates the decontamination results when the PS was applied prior to bacterial inoculation. Both the dark control and light control groups exhibited less than a 1 log reduction in CFU. In contrast, the PDc treatment resulted in 5.61 log reduction. The original population (Co −/−) was 6.94 × 10^7^ CFU cm^−2^, highlighting the difference between the PDc treatment and the controls.

### 3.3. Photodynamic Decontamination of Uneven Surfaces: Strawberries

The surface of strawberries is characterized by embedded achenes that create curvatures and crevices, posing a challenge for effective decontamination.

**Figure 5:**
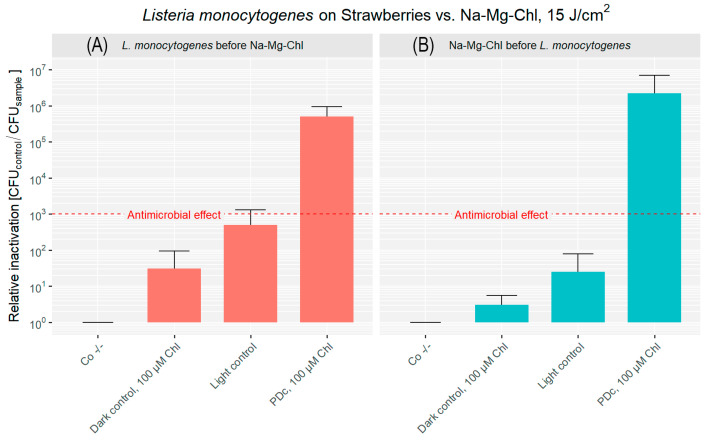
Relative inactivation (CFU_Co −/−_/CFU_sample_) of *L. monocytogenes* after PDc of strawberries. In (**A**), *L. monocytogenes* was applied to the strawberry surface first, followed by the addition of Na-Mg-Chl (Chl on the graph) after 5 min. (**B**) shows Na-Mg-Chl inoculated first, with *L. monocytogenes* added onto the surface after 5 min. PDc was performed using a LED array (395 nm, 17 mW cm^−2^, 15 J cm^−2^). Controls: Co −/− (no light, no PS), Dark control (no light, PS). The light control contained DPBS and was illuminated. PDc was a positive control with 100 µM Na-Mg-Chl and was illuminated. The red dash corresponds to a 3 log reduction. The bars represent the average of six independent biological replicates, including their corresponding error bars.

For strawberries inoculated with *L. monocytogenes* before PDI, the double negative control had an initial bacterial load of approximately 3.22 × 10^6^ CFU cm^−2^ (Figure 5A). Once more, the dark control showed a minimal reduction of CFU. The light control demonstrated a more significant inactivation, achieving over a 2 log reduction. PDc treatment, combining Na-Mg-Chl with light, achieved a substantial inactivation, resulting in a 5.71 log reduction of the bacterial population.

High levels of CFU reduction were observed in strawberries when the bacterial inoculation occurred after Na-Mg-Chl coating, as shown in Figure 5B. Treatment with blue light in combination with Na-Mg-Chl at a concentration of 100 µM resulted in a 6.34 log reduction in bacterial counts. Meanwhile, inoculated controls treated with the PS without illumination showed almost no inactivation. The mean bacterial load without treatment was 5.74 × 10^6^ CFU cm^−2^, indicating approximately one order of magnitude higher bacterial attachment to the strawberry surface compared to apples.

### 3.4. Photodynamic Decontamination of Fuzzy Surfaces: Kiwis

**Figure 6:**
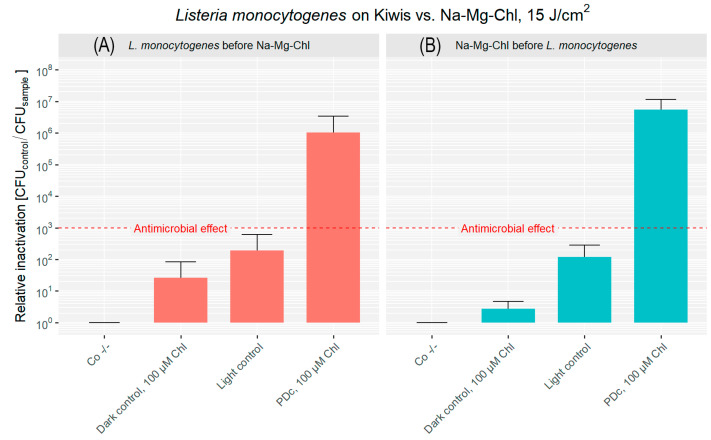
Relative inactivation (CFU_Co −/−_/CFU_sample_) of *L. monocytogenes* after PDc of kiwis. In (**A**), *L. monocytogenes* was applied to the kiwis’ surface, followed by the addition of Na-Mg-Chl after 5 min. (**B**) illustrates Na-Mg-Chl (Chl on the graph) being applied first, with *L. monocytogenes* added to the surface 5 min later. PDc was carried out using an LED array (395 nm, 17 mW cm^−2^, 15 J cm^−2^). Two controls were kept in the dark: Co −/− (DPBS only) and another with the PS and bacteria. The light control contained DPBS and was illuminated. PDc was a positive control with 100 µM Na-Mg-Chl and was illuminated. The red dash indicates a 3 log reduction. The bars represent the average of six independent biological replicates, including their corresponding error bars.

Kiwis were considered the most complex surface. The bacterial attachment to the fuzzy skin or trichomes was comparable to that observed on the other fruits, with a mean of 4.58 × 10^7^ CFU cm^−2^. Figure 6A shows that Photodynamic Decontamination after inoculation with bacteria resulted in a 6.02 log reduction of CFU at the concentration of 100 µM. A slight cytotoxicity was observed in the dark control, while in the light control, a 2 log reduction in phototoxicity was measured.

PDc with the PS applied before bacteria achieved a 6.74 log reduction in bacterial population (Figure 6B). Samples without irradiation showed minimal dark toxicity, while blue light exposure resulted in 2 log reduction. Bacterial attachment was similar, at 1.58 × 10^7^ CFU cm^−2^.

## 4. Discussion

*Listeria monocytogenes* has a significant public health impact. In the US, foodborne illnesses cost around USD 75 billion annually, with listeriosis being particularly costly due to its severity and high hospitalization rates [5,9]. Europe also faces a high fatality rate from listeriosis, making it the second leading cause of foodborne infection deaths after salmonellosis [5]. These serious public health concerns necessitate effective RTE food decontamination methods. PDI is a promising alternative, as it preserves organoleptic properties, is cost-effective, is non-toxic with edible PSs, and is unlikely to induce bacterial resistance [17,18]. Beyond these advantages, compelling evidence has recently confirmed the susceptibility of *L. monocytogenes* to PDI, with studies demonstrating successful inactivation in vitro, on packaging materials, and in both planktonic and biofilm-associated states [24,25,26].

In the first scenario, Na-Mg-Chl was applied after bacterial inoculation, simulating contamination that might occur during early stages such as harvesting or post-harvest storage. The outcomes highlight the efficacy of the PDc, reaching a 5.96 log reduction on apples, a 5.71 log reduction on strawberries, and a 6.02 log reduction on kiwis. It can be stated that there were no significant differences observed across fruit surfaces, indicating that PDI can achieve more than 99.9% photokilling against *L. monocytogenes*, irrespective of challenging fruit surfaces. This is because ROS can reach all crevices on strawberries, and the complex fuzzy surface of kiwis does not inhibit the decontamination of these fruits.

In the second scenario, a simulated coating with Na-Mg-Chl was applied prior to bacterial exposure, representing secondary contamination that may occur during handling, transportation, or further processing steps. Once more, the results underscore the potential of PDI. Apples exhibited a 5.61 log reduction, strawberries achieved a 6.34 log reduction, and kiwis demonstrated the highest inactivation level at 6.74 log units. In this case, PDc was slightly more effective when compared to the first approach. Nevertheless, in general terms, it can be stated that PDc can be achieved in both scenarios with fairly comparable results. While this scenario showed slightly greater effectiveness, the overall findings indicate that PDc can be effectively achieved regardless of whether contamination occurs before or after PS application. From an industrial or agricultural perspective, this consistency suggests that real-world implementation of PDI is feasible across various stages of the supply chain.

In general, Gram-positive bacteria possess a more permeable cell wall when compared to Gram-negative species, which allows for enhanced PS diffusion through the cell structure, enhancing the overall effectiveness of PDI [27,28]. Moreover, Gram-positive species often exhibit elevated production of endogenous porphyrins, such as coproporphyrin and protoporphyrin, which increase their sensitivity to photosensitization-based treatments. In *L*. *monocytogenes*, porphyrins can be endogenously synthesized or induced by 5-aminolevulinic acid (ALA), enabling PDI without exogenous PSs [24,29]. Anionic PSs, such as Na-Mg-Chl, have also been shown to bind efficiently to Gram-positive bacterial surfaces despite their negative charge. Consequently, upon activation of the PSs and formation of singlet oxygen, ROS are generated in close proximity to Gram-positive cells, amplifying the antimicrobial effect [19,21].

When comparing light controls, a higher relative inactivation was observed in strawberries when *L. monocytogenes* was applied first (>2 log reduction). In kiwifruits, the order of bacterial deposition had little influence, with >2 log reductions observed in both application sequences. This consistent inactivation on kiwifruit surfaces may be associated with their characteristic fuzzy surface morphology, although the mechanisms involved remain unclear and warrant further investigation. Overall, it can be hypothesized that some antioxidants were photoactivated under blue light, contributing to the mild antimicrobial effect observed on cut strawberry and kiwi surfaces. Fruit handling and cutting may have facilitated the release of these compounds and their interaction with bacterial cells at the surface, thereby enhancing their photoinduced antimicrobial activity. In addition, this effect may be further enhanced by the endogenous porphyrin synthesis capability of *L. monocytogenes* (as previously discussed). Phenolic compounds such as quercetin, proanthocyanidins, and chlorogenic acid are present in these fruits and have been associated with ROS generation under light exposure [30,31]. In apples, the effect appeared even weaker by comparison, possibly due to the stronger compartmentalization of phenolics within vacuoles or bound to cell walls, limiting their surface availability. In real application, this mechanism is unlikely to be effective, as treatments are typically applied to intact fruits. The limited inactivation observed suggests that an external PS is still required to achieve effective photoinactivation.

Recent studies have demonstrated the effectiveness of PDI as a non-thermal microbial control strategy for fresh produce, highlighting its potential to enhance food safety without compromising quality. For example, in strawberries, curcumin-mediated PDI using blue LED light (420 nm) reduced microbial decay by approximately 20% during storage, chlorophyllin-based PDI achieved a 98% reduction of *L. monocytogenes*, and incorporation of chlorophyllin into chitosan-based edible coatings further enhanced antimicrobial efficacy, with reductions of up to 7 log in vitro and about 1.4 log on the fruit surface, including pathogens such as *Salmonella enterica* and spoilage fungi. Importantly, across these studies, PDI treatments did not adversely affect produce firmness, color, antioxidant activity, or visual quality, while also extending strawberry shelf-life by up to three days [32,33,34]. In apples, curcumin-based PDI effectively inactivated *Escherichia coli* while also preventing browning and weight loss during storage [28]. In kiwis, PDI has been shown to inactivate *Pseudomonas syringae* pv. *actinidiae* in pollen, reducing the risk of pathogen transmission [35].

Current studies have also examined the approach of incorporating PSs into packaging materials. For instance, chitosan-riboflavin composite films applied superficially have demonstrated ≥ 3 log reduction of *L. monocytogenes* on salmon [17,36]. Similarly, embedding PSs in carboxymethyl cellulose films with polyphenol extract and carbon dots derived from coffee husk has been shown to extend the shelf life of fresh-cut apples up to 7 days [37]. Considering that wax depletion increases water loss and microbial vulnerability during storage, particularly noted in studies on apple cultivars [38], embedding PSs in coatings could enable a dual-function approach, providing both a physical moisture barrier and light-activated antimicrobial activity. These studies highlight the promise of packaging-mediated PS delivery as a complementary approach to direct application. Future research should therefore prioritize developing hybrid systems to enhance both food safety and shelf life.

## 5. Conclusions

This study demonstrates that Photodynamic Inactivation (PDI) using sodium-magnesium-chlorophyllin and blue LED light is a highly effective strategy for reducing *Listeria monocytogenes* on fresh fruits with diverse surface characteristics. Substantial log reductions (5 to 6.7 log units) were consistently achieved on apples, strawberries, and kiwis, independent of whether the photosensitizer was applied before or after bacterial contamination. These results confirm that PDI is robust against variations in fruit surface texture and contamination sequence, reflecting realistic scenarios along the food supply chain.

Moreover, the ability of PDI to deliver significant microbial reductions without compromising product quality highlights its promise as a non-thermal, consumer-acceptable intervention to enhance the microbiological safety of fresh produce. Nevertheless, the consistency and reliability of this approach under real-world food processing conditions must be verified in future studies in collaboration with industrial partners. Further investigations should also address the impact of naturally occurring mixed microbial communities on PDI efficacy, as such interactions could influence treatment outcomes under practical conditions.

In addition, consumer acceptance, regulatory aspects, and potential labeling requirements related to PDI application should be explored during the transition from laboratory research to industrial implementation. While the use of sodium-magnesium-chlorophyllin, approved as the food additive E140, suggests a feasible regulatory pathway, future development and assessment by experts in regulatory affairs will be essential to ensure compliance and public confidence. Overall, the adoption of this technology could play a pivotal role in mitigating public health risks and economic losses associated with foodborne pathogens, supporting safer and more sustainable food systems.

## Data Availability

The original contributions presented in this study are included in the article/Supplementary Materials. Further inquiries can be directed to the corresponding author.

## Supplementary Materials

The following supporting information can be downloaded at: https://www.mdpi.com/article/10.3390/microorganisms14010059/s1, Table S1: Relative inactivation of apples inoculated with *L. monocytogenes* before Na-Mg-Chl, Table S2: Relative inactivation of apples previously coated with Na-Mg-Chl before *L. monocytogenes* inoculation, Table S3: Relative inactivation of strawberries inoculated with *L. monocytogenes* before Na-Mg-Chl application, Table S4: Relative inactivation of strawberries previously coated with Na-Mg-Chl before *L. monocytogenes* inoculation, Table S5: Relative inactivation of kiwis inoculated with *L. monocytogenes* before Na-Mg-Chl application, Table S6: Relative inactivation of kiwis previously coated with Na-Mg-Chl before *L. monocytogenes* inoculation.

## Abbreviations

The abbreviations used throughout this manuscript are as follows:

ALA	5-aminolevulinic acid
CFU	Colony forming unit(s)
Co −/−	Double negative control (no light, no PS)
DPBS	Dulbecco’s phosphate-buffered saline
*L. monocytogenes*	*Listeria monocytogenes*
MIC	Minimum inhibitory concentration
Na-Mg-Chl	Sodium-Magnesium-Chlorophyllin
PDc	Photodynamic decontamination of food
PDI	Photodynamic Inactivation
PS	Photosensitizer
ROS	Reactive oxygen species
RTE	Ready to eat
