# Supplementary material for: Photodynamic Decontamination of Food: Assessing Surface Challenges Against Listeria monocytogenes
Source: Microorganisms. 2025 Dec 26;14(1):59. doi: 10.3390/microorganisms14010059 (PMC12843728; doi:10.3390/microorganisms14010059)
Supplement: Supplementary file 1 [file microorganisms-14-00059-s001.zip › microorganisms-4003442-supplementary.pdf]

Supplementary Information

Table S1. Relative inactivation of apples inoculated with *L. monocytogenes* before Na-Mg-Chl application (n = 6).

	**Relative inactivation [$CFU_{control}$/CFU_{sample}]**		
	**mean**	±	SD
Co -/-	1.00E+00	±	0.00E+00
Dark control, 100 µM Chl	9.21E+00	±	2.07E+01
Light control	4.75E+00	±	8.11E+00
PDc, 100 µM Chl	9.12E+05	±	1.15E+06

Table S2. Relative inactivation of apples previously coated with Na-Mg-Chl before *L. monocytogenes* inoculation (n = 6).

	**Relative inactivation [$CFU_{control}$/CFU_{sample}]**		
	**mean**	±	SD
Co -/-	1.00E+00	±	0.00E+00
Dark control, 100 µM Chl	1.90E+00	±	1.83E+00
Light control	6.49E+00	±	1.13E+01
PDc, 100 µM Chl	4.09E+05	±	6.65E+05

Table S3. Relative inactivation of strawberries inoculated with *L. monocytogenes* before Na-Mg-Chl application (n = 6).

	**Relative inactivation [$CFU_{control}$/CFU_{sample}]**		
	**mean**	±	SD
Co -/-	1.00E+00	±	0.00E+00
Dark control, 100 µM Chl	3.05E+01	±	6.28E+01
Light control	4.96E+02	±	8.21E+02
PDc, 100 µM Chl	5.07E+05	±	4.49E+05

Table S4. Relative inactivation of strawberries previously coated with Na-Mg-Chl before *L. monocytogenes* inoculation (n = 6).

	**Relative inactivation [$CFU_{control}$/CFU_{sample}]**		
	**mean**	±	SD
Co -/-	1.00E+00	±	0.00E+00
Dark control, 100 µM Chl	3.02E+00	±	2.49E+00
Light control	2.50E+01	±	5.34E+01
PDc, 100 µM Chl	2.23E+06	±	4.69E+06

Table S5. Relative inactivation of kiwis inoculated with *L. monocytogenes* before Na-Mg-Chl application (n = 6).

	**Relative inactivation [$CFU_{control}$/CFU_{sample}]**		
	**mean**	±	SD
Co -/-	1.00E+00	±	0.00E+00
Dark control, 100 µM Chl	2.65E+01	±	5.71E+01
Light control	1.93E+02	±	4.20E+02
PDc, 100 µM Chl	1.04E+06	±	2.40E+06

Table S6. Relative inactivation of kiwis previously coated with Na-Mg-Chl before *L. monocytogenes* inoculation (n = 6).

	**Relative inactivation [$CFU_{control}$/CFU_{sample}]**		
	**mean**	±	SD
Co -/-	1.00E+00	±	0.00E+00
Dark control, 100 µM Chl	2.72E+00	±	1.93E+00
Light control	1.18E+02	±	1.62E+02
PDc, 100 µM Chl	5.55E+06	±	5.88E+06
